# Joint Diagnosis of Pneumonia, COVID-19, and Tuberculosis from Chest X-ray Images: A Deep Learning Approach

**DOI:** 10.3390/diagnostics13152562

**Published:** 2023-08-01

**Authors:** Mohammed Salih Ahmed, Atta Rahman, Faris AlGhamdi, Saleh AlDakheel, Hammam Hakami, Ali AlJumah, Zuhair AlIbrahim, Mustafa Youldash, Mohammad Aftab Alam Khan, Mohammed Imran Basheer Ahmed

**Affiliations:** 1 Department of Computer Engineering, College of Computer Science and Information Technology, Imam Abdulrahman Bin Faisal University, P.O. Box 1982, Dammam 31441, Saudi Arabia; 2 Department of Computer Science, College of Computer Science and Information Technology, Imam Abdulrahman Bin Faisal University, P.O. Box 1982, Dammam 31441, Saudi Arabia

**Keywords:** deep learning, chest X-ray, joint diagnosis, radiology, convolution neural network (CNN), pneumonia, tuberculosis, COVID-19

## Abstract

Pneumonia, COVID-19, and tuberculosis are some of the most fatal and common lung diseases in the current era. Several approaches have been proposed in the literature for the diagnosis of individual diseases, since each requires a different feature set altogether, but few studies have been proposed for a joint diagnosis. A patient being diagnosed with one disease as negative may be suffering from the other disease, and vice versa. However, since said diseases are related to the lungs, there might be a likelihood of more than one disease being present in the same patient. In this study, a deep learning model that is able to detect the mentioned diseases from the chest X-ray images of patients is proposed. To evaluate the performance of the proposed model, multiple public datasets have been obtained from Kaggle. Consequently, the proposed model achieved 98.72% accuracy for all classes in general and obtained a recall score of 99.66% for Pneumonia, 99.35% for No-findings, 98.10% for Tuberculosis, and 96.27% for COVID-19, respectively. Furthermore, the model was tested using unseen data from the same augmented dataset and was proven to be better than state-of-the-art studies in the literature in terms of accuracy and other metrics.

## 1. Introduction

Healthcare is among the most important sectors in human lives as well as the economic system. It has been proved that there is a positive association between a booming healthcare sector and a thriving economy [1]. Artificial intelligence has been playing a major role in the advancement of the healthcare sector in past several years. This is due to the growing availability of the clinical data that are used in training these systems. These data are consequently used for detecting, predicting, or finding the best possible treatments for various diseases. This study proposes a deep learning model to detect and diagnose pneumonia, COVID-19, and tuberculosis using chest X-ray images of infected patients. Tens of millions of people die due to lung diseases in the United States alone. The lungs form a complex system, and different diseases hit different areas of this system. For instance, some diseases, like asthma, affect the airways of the lungs, causing them to be inflamed, which causes shortness of breath in the host. Moreover, diseases like pneumonia, tuberculosis, and lung cancer affect the air sacs inside the lungs, which are called alveoli [2]. In this study, we built a deep learning model covering three diseases that affect the air sacs in the lungs. These diseases are pneumonia, COVID-19, and tuberculosis. 

Pneumonia is an infectious disease that fills alveoli with fluid and pus, causing the patient to experience shortness of breath and/or painful breathing. Additionally, pneumonia is the leading infectious cause of death in children under five years [3]. 

COVID-19 is an airborne infectious disease that emerged at the end of the year 2019 in the city of Wuhan, China. The disease quickly spread around the world, infecting over 600 million people while causing the death of over 6.5 million people as of September 2022 [4]. In Saudi Arabia alone, the disease infected over 800,000 people while killing 9000 people [5]. 

Tuberculosis (TB) is the leading infectious disease worldwide, killing over 1.5 million people each year. It is caused by bacteria called Mycobacterium tuberculosis, and it affects the alveoli. Most deaths caused by TB are in low- and middle-income countries [6]. 

A definitive way of diagnosing all the previously mentioned diseases is by analyzing X-ray imagery of patients carrying these diseases. Hence, in this study, we built a model, using deep learning methods, that can detect these diseases preemptively in hopes of diagnosing patients early, which will give them a higher chance of survival or being treated, and aiding radiologists’ decision making. 

Moreover, the joint diagnosis of these diseases is crucial for improving accuracy and efficiency in diagnosing patients who may have multiple diseases, particularly during a pandemic like COVID-19. In this regard, several studies have been conducted in literature with their own pros and cons. For instance, previous studies have encountered challenges such as small or biased datasets, which this study seeks to address using a larger and more diverse dataset. Further, the study provides a comprehensive review of recent studies using deep learning techniques for the joint diagnosis of pneumonia, COVID-19, and tuberculosis from chest X-ray images, highlighting areas of success and limitations, and proposing potential directions for future research. To accomplish this, in this study, a convolutional neural network (CNN) multiclass model was developed to detect pneumonia, COVID-19, and tuberculosis in chest radiographs. The study had four main steps. First, the data were split into 80% for training and 20% for testing. Second, the images were resized into 300 × 300. In the third step, a CNN was built with the following classes: COVID-19, No-findings, Pneumonia, and Tuberculosis. The final step was to evaluate the model’s performance using accuracy, recall, and precision. 

The rest of the paper is structured as follows: Section 2 contains a review of the related literature; Section 3 introduces the dataset and its features. Section 4 contains the methodology, and Section 5 presents the results, while Section 6 concludes the paper.

## 2. Review of the Literature

In this section, we discuss the studies in the literature that have been conducted on the early diagnosis and detection of the lung diseases chosen in this study. The following sections cover machine learning and deep learning approaches for pneumonia, COVID-19, and tuberculosis, respectively, while the last section is dedicated to studies with more than one mentioned disease.

### 2.1. Pneumonia

Hashmi et al. [7] proposed an efficient model for detecting pneumonia that is trained on chest X-ray images using a weighted classifier that combines weighted predictions from models such as ResNet18, Xception, DenseNet121, and more. Transfer learning is used to fine-tune the results of the models to obtain higher validation accuracy. The weighted classifier was able to achieve a test accuracy of 98.43% on unseen data. Stephen et al. [8] developed a model to detect pneumonia in chest X-ray images by identifying and classifying the disease using a CNN. To increase the performance of the model, parameter and hyperparameter were laboriously tuned. The best results achieved by the model are a training accuracy of 94.81% and a validation accuracy of 93.73%. ElShennawy et al. [9] established a CNN model to detect pneumonia using chest X-ray images and using ResNet152V2 and MobileNetV2 as extraction models. A public dataset that contained normal and infected chest X-ray images was used. The best outcome obtained by the CNN model is 99.22% accuracy. Szepesi et al. [10] developed transfer learning models such as InceptionV3 and ResNet50, using a dataset that contains normal and pneumonia-infected children’s chest X-ray images. ResNet50 obtained an accuracy of 89.06%, and InceptionV3 achieved a higher accuracy of 90.94%. Qaimkhani et al. [11] proposed a deep learning technique to identify lung disease at an early stage by using medical imaging. Disease identification and classification are widely carried out with CNN. In addition, features learned by CNN models on large-scale datasets can be applied to image classification tasks as well. For the classification of abnormal and normal chest X-rays, pre-trained CNN models were used as feature extractors followed by different classifiers. Each image type (pneumonia/normal) is divided into its own subdirectory (train, test, and validation). Pneumonia and normal X-ray photos comprise 5863 JPEG images. This study uses three deep learning models: ANN, CNN, and VGG19 and achieved the highest accuracy of 97% using VGG19. 

Another celebrated study in [12] presented a machine learning framework for pneumonia detection from chest X-ray images comprising dense CNN-160, ResNet-121, and VGG-16 ensemble models. With 97.69% accuracy, 100% recall, and 0.9977 area under the curve scores, the scheme was promising for multivariate classification of normal, bacterial, and viral pneumonia in chest X-ray images.

### 2.2. COVID-19

Ramadhan et al. [13] built a VGG16-CNN to classify three public datasets that consist of chest X-ray images of COVID-19 patients. Binary classification was conducted on three datasets, and the model achieved 97% accuracy on the first dataset, 98.73% on the second dataset, and the highest accuracy was achieved using the third dataset, i.e., 99.76%. Jain et al. [14] used deep learning-based CNN models like Xception, InceptionV3, and ResNeXt and compared their performances on detecting COVID-19 from chest X-ray images. The Xception model gave the best accuracy of 97.97%.

Hussain et al. [15] developed a CNN model called CoroDet and used it on three different classifications levels as 2, 3, and 4 classes. Using a dataset containing chest X-rays of COVID-19 patients, the two-class (binary) classification achieved 99.1% accuracy as the highest, followed by 92.4% on the three-class classification, and lastly 91.2% for the four-class classification. Nayak et al. [16] used deep learning-based CNN models such as GoogleNet, SqueezeNet, and VGG-16. Experiments were performed using a dataset containing COVID-19 patient X-ray images from various sources; GoogleNet reached 98.62% accuracy, 96.15% was achieved by VGG-16, and AlexNet attained the best accuracy with 99.05% accuracy. 

Zagrouba et al. [17] proposed a supervised machine learning model for the modelling and simulation of the COVID-19 outbreak. A support vector machine (SVM)-based forecasting approach has been investigated that resulted in 98.88% and 96.79% accuracies during training and testing phases, respectively. Similarly, Rahman et al. [18] proposed a supervised machine learning-based predictive model for the COVID-19 outbreak. SVM-based multi-fold cross validation technique has been investigated. The scheme was promising in terms of achieving a high validation accuracy of 98.4%. Ahmed et al. [19] proposed a fuzzy rule-based system (FRBS) for an early identification of COVID-19 using clinical data. The model exhibited prediction accuracy, precision, sensitivity, specificity, and f1-score as 88.78%, 72.22%, 68.42%, 93.67%, and 69.28%, respectively. Naqvi et al. [20] presented a comprehensive study to express the gross damages produced by the coronavirus around the globe in terms of global healthcare, public safety, economics, industry, businesses, travelling restriction, and several other factors. 

Similarly, Nasiri and Hasani [21] proposed a deep learning approach using DenseNet169 deep neural network (DNN) in coordination with extreme gradient boosting (XGBoost) algorithm for fine-tuned classification of X-ray images as COVID-19 positive. The scheme was promising in terms of accuracy, specificity, and sensitivity. Nonetheless, binary classification outperformed tertiary classification in all the evaluation metrics. Furthermore, the analyses exhibited 98.23% and 89.70% accuracy, 99.78% and 100% specificity, and 92.08% and 95.20% sensitivity in binary and ternary problems, respectively. 

Khan et al. (2022) [22] proposed a new channel boosted CNN approach to COVID-19 detection from chest X-ray images. In this regard, they investigated a new idea of split–transform–merge (STM) equipped with region and edge-based (RE) operation. Consequently, they coined the scheme as STM-RENet. The scheme was implemented over three datasets, and the best accuracy achieved was 96.53%, and F-score was 95%.

### 2.3. Tuberculosis (TB)

Heo et al. [23] aimed to use CNN to detect TB in chest X-rays from annual workers health examinations data and compared it with demographic added CNN. Both models were trained on 1000 X-ray images of both positive and negative cases of TB. Feature extraction was conducted using VGG19, ResNet50, and others. Age, height, and gender were recorded as demographic variables. The best area under the cover (AUC) was obtained using VGG19 with 90.75% for CNN with images only, and 92.13% with demographic variables added. Hwa et al. [24] presented an approach using ensemble deep learning to detect TB using chest X-ray images and canny edge detected images. Two sets of features were extracted, the first one from the X-ray images, and the second set was extracted from the canny edge detected images. The best results obtained by the ensemble model in terms of accuracy was 89.77%, and sensitivity of 90.91%. 

Rahman et al. [25] proposed a set of nine different Deep CNNs (ResNet18, ResNet50, ResNet101, ChexNet, InceptionV3, VGG19, DenseNet201, SqueezeNet, and MobileNet) used for transfer learning from their pre-trained initial weights, and have been trained, tested, and validated to detect TB cases. Several public datasets containing lung X-ray images of normal and infected patients were used. The best accuracy gained was 98.7% using DenseNet201. Iqbal et al. [26] developed a deep learning-based framework called TBXNet capable of properly classifying a vast quantity of TB chest X-rays images. It was performed using three public datasets containing chest X-ray images of infected and normal patients. The framework achieved 99.17% for dataset-A containing binary classes. Followed by 98% accuracy obtained from dataset-B containing three classes and lastly dataset-C with four classes gained 95.1% accuracy. 

Norval et al. [27] used CNN to detect pulmonary TB in patients using their chest X-ray images. A hybrid approach using the original statistical computer-aided detection method combined with neural networks was also investigated. Simulations have been performed on 406 normal images and 394 abnormal images. X-ray images for the dataset were collected from Shenzhen number 3 Hospital in Shenzhen, Guangdong province, China. The dataset contained images in JPEG format. There were 336 normal and 326 abnormal X-rays showing various manifestations of TB. The input data was enhanced and then the simulation was executed. Hybrid methods resulted in the highest accuracy of 92.54%.

### 2.4. Joint Diseases Detection Studies

Bhandari et al. [28] presented a joint study for classification of chest X-ray images into possibly COVID-19, pneumonia, and TB using deep learning as well as an Explainable Artificial Intelligence (XAI) framework. The dataset comprised 7132 chest X-ray images. The study was able to achieve an average test accuracy of 94.31 ± 1.01% and validation accuracy of 94.54 ± 1.33 for 10-fold cross-validation.

Venkataramana et al. [29] proposed a multi-level classification system that contains two models. The first model is a binary classification model that classifies TB and pneumonia. The second model is for detecting the types of pneumonia by considering the output of the first model as an input. Synthetic minority oversampling technique (SMOTE) was used to balance the classes of the dataset, which comprised 14,693 images. The study was able to achieve an accuracy of 95.7% before balancing and achieved 96.6% after balancing. 

Hasan et al. [30] presented a model to detect pneumonia in COVID-19 patients. A CNN model was used as a feature extractor and a classifier and VGG16 was used for training the model. Multiple machine learning tools were used such as LabelBinarizer to perform one-hot encoding on labeled X-ray images. The study achieved an average accuracy of 91.69% and 95.92% sensitivity in predicting pneumonia. 

Ibrokhimov and Kang [31] developed a deep learning diagnosis system for detecting pneumonia using X-ray images. Transfer learning was employed using pretrained models such as VGG19 and ResNet50. The dataset comprised 11,956 COVID-19 samples, 11,263 viral or bacterial pneumonia, and 10,701 normal samples. The VGG19 model outperformed the ResNet50 by scoring an average accuracy of 96.6% across all classes. 

Bashar et al. [32] proposed a deep learning approach to diagnose COVID-19 and pneumonia using X-ray images. In this regard, a public dataset containing 21,165 chest X-ray images was obtained from Kaggle. Image enhancements and data augmentation methods as well as multiple transfer learning algorithms such as VGG16, VGG19, GoogleNet, and more were used in this study. The highest accuracy achieved as 95.63% using the VGG16 algorithm on the augmented and enhanced dataset. Baltazar et al. [33] proposed COVID-19 and pneumonia detection models by generating their own data. They optimized five different deep learning architectures, namely, InceptionV3, InceptionResNetV2, Xception, VGG, and MobileNet, and evaluated their diagnostic performance using various evaluation metrics. In this study, InceptionV3 achieved the highest results with 86% sensitivity, 99% specificity, and 91% positive predictive value (PPV), respectively. A similar approach was proposed in [34] where the authors proposed a fusion of deep features and light gradient boosting machine (LightGMB) to detect COVID-19 from chest X-ray images. In this regard, a dataset of 1125 images was used, and the proposed scheme exhibited 98.54% and 91.11% accuracies in the two-class (COVID-19, Healthy) and three-class (COVID-19, Healthy, Pneumonia), respectively. The authors aimed to investigate a gradient-weighted class activation mapping (Grad-CAM) as a futuristic analysis approach in the respective study. 

Likewise, authors in [35] presented a deep learning model for joint diagnosis of COVID-19 and pneumonia using chest X-ray images. In this regard, they investigated various image processing approaches like weber local descriptor and local binary patterns [36], contextual style transfer [37], and generalization of intensity distribution [38]. The scheme obtained a decent accuracy for binary and multivariate classification as 91.5% and 91.11%, respectively.

A summary of literature review is presented in Table 1 containing studies employed for multiple/joint disease detection from chest X-ray images. The highest average accuracy achieved in the validation phase for multiple disease is obtained by [29] as 96.6% with SMOTE and deep learning as a four-class problem. Nonetheless, as far as the binary classification problems are concerned, the highest accuracy achieved by [34] was 98.54%. That is quite understandable as, in case of binary classification, classes are far from each other (separable), hence, more interclass difference and less vulnerability to misclassification. While in the case of multiclassification, it is less prone to misclassification error. That indicates further improvement in the accuracy of the joint models which is the target of the proposed study where a four-class problem has been addressed.

## 3. Dataset

This study used three different datasets of chest radiographs (X-ray images) that are publicly available on Kaggle. The COVID-19 radiography database [39] was used for classifying COVID-19, and it contains 3616 COVID-19 chest X-rays, and 10,192 of normal radiographs used for classifying ‘NO-FINDINGS’ class. For pneumonia, we used ‘Chest X-ray Images (Pneumonia)’ dataset on Kaggle [40]. The dataset contains 4273 pneumonia chest radiographs. Finally, for tuberculosis, we used ‘Tuberculosis (TB) Chest X-ray Database’ [41] created by a team of researchers from Qatar university and the university of Dhaka. The database contains 3500 tuberculosis chest radiographs. 

**Figure 1:**
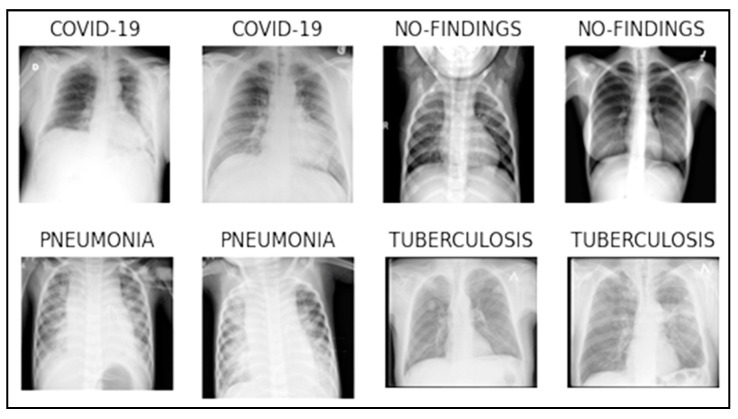
Random sample of images from each class.

The term “database” in this context refers to the integration of distinct datasets collected from various reputable data sources, such as Kaggle and IEEE Dataport. The criteria behind the dataset selection were mainly based on recent studies investigated and commonly used datasets for a comprehensive analysis. Table 2 provides the images distribution among the classes in the dataset. Figure 1 depicts few sample images from the augmented dataset with and without a disease.

## 4. Methodology

**Figure 2:**
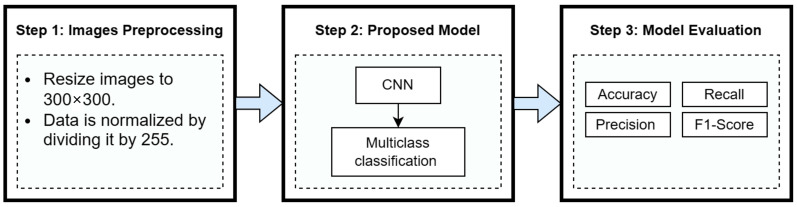
Study’s main steps.

This study is investigating a convolutional neural network to detect COVID-19, pneumonia, and tuberculosis using chest radiographs. The study is divided into three main steps as seen in Figure 2 and presented subsequently.

### 4.1. Image Preprocessing

In this step, all images were resized into 300 × 300 dimensions. Resizing the images is a necessary step before feeding the data into the proposed convolutional neural network. The dimension was chosen since most of the data were equal to or greater than 300 × 300. In other words, the reason for resizing the images to 300 × 300 dimensions is that this is the smallest size in a couple of the datasets being used in this study. The main steps involved in image preprocessing are image resizing, denoising, normalization and filtering. Consequently, a homogeneous and coherent dataset is obtained despite the heterogenous sources being involved in data collection.

### 4.2. Proposed Model

**Figure 3:**
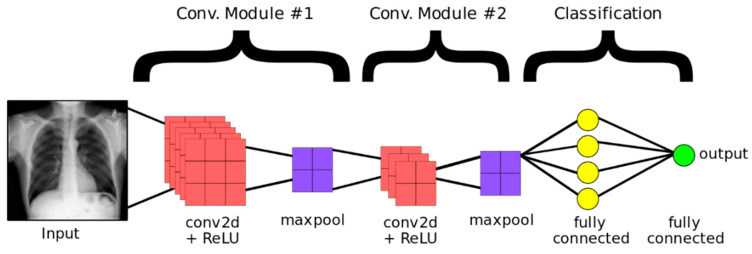
CNN architecture example.

In literature, convolutional neural networks have been successfully applied to a broad range of image processing applications, especially medical imaging. Medical images, such as chest radiographs, are important to diagnose various lung diseases. CNNs are very useful for finding new features and patterns that can aid radiologists’ decision making when diagnosing. In this study, we are proposing a convolutional neural network that takes a grayscale image as an input, with dimensions (300, 300, 3). The model consists of five blocks of convolutional layers. The first block contains one convolutional layer with 16 channels and a ReLU activation function. This layer is followed by a max-pool layer to aggregate the first block outcomes. The second block contains one convolutional layer with 32 channels and a ReLU activation function, followed by a max-pool layer. In the third block, one convolutional layer with 64 channels with ReLU activation function, followed by a max-pool layer. The last two convolutional blocks consist of two convolutional layers, one with 128 channels and the other 256 channels. Each is followed by a max-pool layer. The said configuration was obtained after several trials. Throughout the training phase, we progressively incorporated convolutional blocks until the desired outcomes were achieved. Additionally, we iteratively introduced and removed dropout layers between the convolutional blocks, while adjusting the dropout rate, to find the optimal equilibrium that maximizes the desired results. Moreover, the proposed architecture has been obtained by a hit and trial method and after certain trial and error, the architecture was finalized. As described the proposed architecture is presented in Figure 3 and Table 3 enlists the set of parameters used in the study.

**Figure 4:**
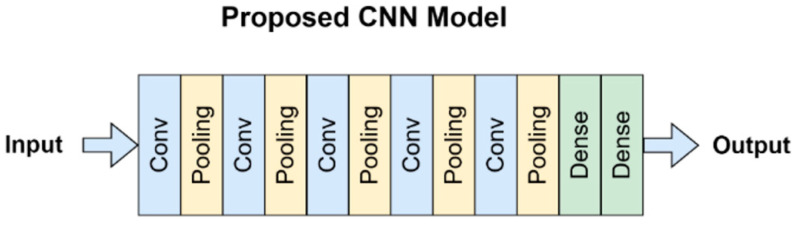
Proposed model architecture.

Figure 4 shows the architecture of the proposed CNN model as explained already. 

### 4.3. Model Evaluation

To evaluate the proposed model, several evaluation metrics, like accuracy, precision, recall, and F1-score, are used. Also, a confusion matrix and a classification report have been used to evaluate the model. Additionally, at the inference stage, the model was evaluated using 100 images of unseen data taken from the same database, and model was trained on 25 images from each class. The most widely used evaluation metrics for similar kinds of studies are given in Equations (1)–(4) in their mathematical form [42,43,44,45,46].

(1) $$Accuarcy=\frac{TP+TN}{TP+TN+FP+FN}$$

(2) $$Precision=\frac{TP}{TP+FP}$$

(3) $$Recall=\frac{TP}{TP+FN}$$

(4) $$F1\text{-}Score=2\times \frac{Precision\times Recall}{Precision+Recall}$$

True Positive (*TP*) represents the number of correct predictions that an instance is positive, whereas True Negative (*TN*) is the number of correct predictions that an instance is negative. False Positive (*FP*) represents the number of incorrect predictions that an instance is positive, whereas False Negative (*FN*) represents the number of incorrect predictions that an instance is negative.

#### 4.3.1. Welch’s *t*-Test

Welch’s *t*-test is used to test the hypothesis that two populations have equal means and unequal variances, also known as the unequal variance *t*-test [47]. In the current study, it is reasonable to conduct this test since the classes are not balanced (Table 2). 

Since the test is applicable on the two populations, we summed up the diseases as single population with 11,389 instances and the no-finding class with 10,192 instances. Upon calculation [48], the obtained t value as t = 9.314913. Because the absolute value of the test statistic 6.421 was not larger than the obtained t value, the null hypothesis of the test cannot be rejected. Hence, there is not sufficient evidence to state that the mean values of the two considered populations are significantly different. Same was observation of the authors during data preprocessing phase.

#### 4.3.2. Limitations of the Study

As far as the limitations of the study are concerned, one of the primary limitations in the proposed model is imbalanced class instances. Furthermore, it is worth noting that the model’s generalizability may be limited when applied to images sourced from diverse datasets.

## 5. Results

In this section, we will discuss the results achieved by the experimental studies of the proposed multiclass model. 

**Figure 5:**
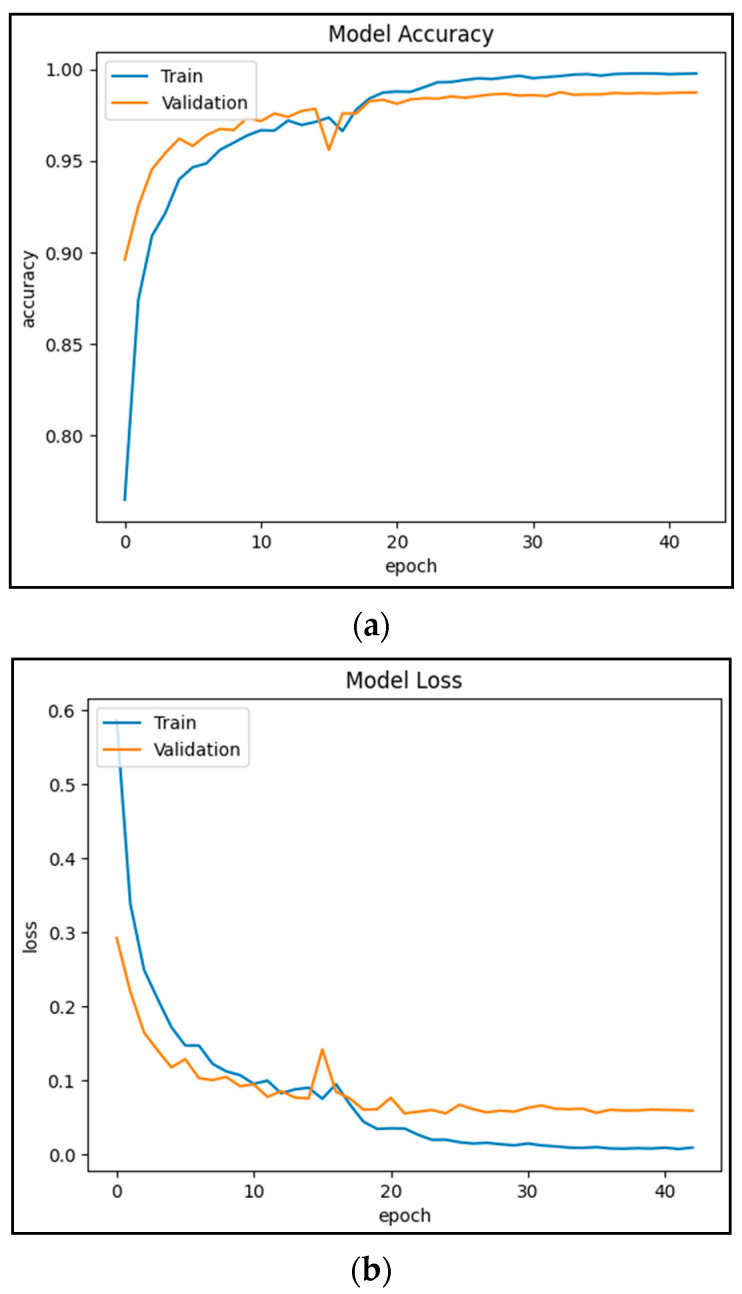
(**a**) Model’s accuracy plot; (**b**) Model’s loss plot.

Figure 5a presents the average model accuracy with respect to the number of epochs. Roughly after 30 epochs, the system reaches the steady state when the transients are over. The model achieved a 98.72% accuracy in validation phase while nearly 100% accuracy is obtained in the training phase, which is quite encouraging.

Similarly, Figure 5b presents the average miss rate with respect to the number of epochs. After 30 epochs, the miss rate in the training approaches almost zero while the miss rate in validation approaches 1.28%, which is quite acceptable. 

Similarly, Table 4 enlists other evaluation metrices that are precision, recall, and F1-score for each class, respectively. It is noteworthy that Pneumonia class obtains the highest values of precision, recall, and F1-score, i.e., 99.89%, 99.66%, and 99.77%, respectively. This might be due to the nature of the classification features related to the disease and a greater number of instances compared to the other two disease classes, respectively.

The following class with the highest precision of 98.90% is Tuberculosis. But it has a relatively less recall and F1-score of 98.10% and 98.50%, respectively. However, the class with label No-Findings obtained slightly lower precision of 98.72% than Tuberculosis but with better recall and F1-score values of 99.35% and 99.04%, respectively. That is understandable because it contains instances very much comparable to the collective instances of the three diseases.

The COVID-19 class obtained relatively poor evaluation metrics values as 97%, 96.27%, and 96.63% for precision, recall, and F1-score, respectively. This could be due the reason that in the dataset, number of instances for COVID-19 were relatively lower than other classes. Nonetheless, wholistically, all evaluation metrics values for all the classes are above 96.27%, which is quite promising in contrast to joint disease studies in the literature. 

**Figure 6:**
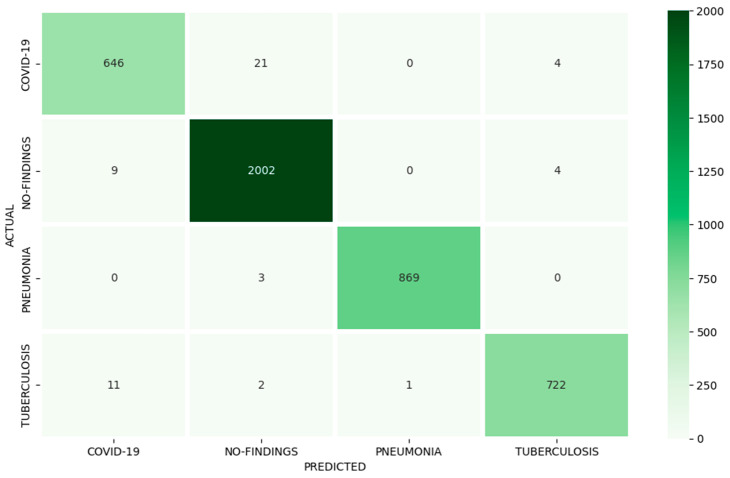
Proposed model confusion matrix.

As a further discussion, probably the Pneumonia class had the best quality of images of all the classes and consequently the features that helped it obtain the best accuracy, recall, and F1-score by using the proposed deep learning model in contrast to other classes. As for COVID-19, it could be that the reticular pattern typical of COVID-19 can be difficult to detect on chest X-ray. Moreover, the resemblance between COVID-19 images and the No-findings class led to numerous misdiagnoses, thereby diminishing the overall accuracy of the COVID-19 class. Figure 6 presents the confusion matrix for the proposed multiclass classification problem.

Now, it is the right time to discuss the results of the proposed model for the inference stage in which we tested the model using random and unseen data obtained from the same database that the model has been trained on. In this regard, we randomly selected 25 images from each class with equal probability and diagnosed them using the proposed trained model. We can observe the results in Table 5. In this stage, it is apparent that all the classes obtained 100% classification accuracy except the COVID-19 class with 88% classification accuracy and 12% miss prediction. The results are like those presented in Table 4 in terms of other evaluation metrics such as precision, recall, and F1-score.

### Comparison with State-of-the-Art

In this section, we compared the proposed model with the state-of-the-art techniques in the literature. The schemes have been chosen based on dataset type (chest radiographs), number, and type of the diseases predicted in the model. Table 6 presents the comparison with four studies Bhandari et al. [26], Venkataramana et al. [28], Bashar et al. [31], and Liu et al. [41], respectively. Studies in [27,28] have same number of classes as of the proposed study that are COVID-19, Pneumonia, Tuberculosis, and No-Finding. Proposed scheme outperforms both schemes with an average validation accuracy of 98.72% followed by [28] with 96.6% with balanced dataset and [27] with 94.54 ± 1.33%, respectively.

Further, the scheme was compared with two more schemes that are [31] with the same sized dataset but with one different type of lung disease and [41] with three classes of normal, COVID-19, and Pneumonia. The proposed scheme again outperforms in terms of average validation accuracy of 98.72% in contrast to 91.11 in [40], 95.63% in [31], and 91.11% in [41], respectively.

## 6. Conclusions

COVID-19, pneumonia, and tuberculosis are among the most dangerous and fatal lung diseases. In this study, we developed a multiclass convolutional neural network that detects the mentioned diseases in chest radiographs (X-rays) with the highest average validation accuracy of 98.72%. The model can be used as a clinical decision support system for healthcare experts. The model’s development phase had three main steps, namely, preprocessing, model building, and finally, evaluation. The analyses were performed on the dataset that comprised 21,581 chest X-ray images obtained from various well-known public sources. The scheme outperformed the similar state-of-the-art techniques in the literature in terms of average accuracy. The analyses are conducted on a relatively imbalanced dataset that resulted in a slightly lower accuracy in COVID-19 class due relatively smaller number of instances compared to the other classes. This is the major limitation of the study that makes it vulnerable to slight misclassification in certain classes/diseases only. In future, the authors intend to investigate the effects of dataset balancing techniques such as SMOTE on the proposed model. It can be safely forecasted that balancing the dataset prior to training the model may further improve the results in terms of average accuracy of the model. Moreover, it is greatly emphasized to use the concept of transfer learning and pretrained models to further fine-tune the proposal and make it robust against potentially diverse datasets investigated in the literature [49,50].

## Figures and Tables

**Table 1:** Literature summary of joint disease detection studies.

Study	Dataset Size	Diseases/ Classes	Techniques Used	Advantages	Limitation
[21]	Cohen dataset and ChestX-ray8 1125 X-ray images	Binary (COVID-19/Healthy) Ternary (COVID-19, Healthy/Pneumonia)	Deep Learning and XGBoost	Good accuracy for binary classification 98.23% for binary 89.70% for ternary	Ternary classification needs improvement. Only binary and ternary cases
[28]	Public dataset with 7132 chest X-ray images	COVID-19 Pneumonia Tuberculosis No-Finding	Deep Learning and XAI	Good validation accuracy Testing: 94.31 ± 1.01% Validation: 94.54 ± 1.33%	Results are based on a smaller subset of dataset
[29]	Public dataset with 14,693 chest X-ray images	COVID-19 Pneumonia Tuberculosis No-Finding	SMOTE and Deep Learning	Considerable accuracy with balancing the dataset. 95.7% without Balancing 96.6% with Balancing	Annotated dataset with already two diseases and COVID-19 was predicted
[32]	Public dataset with 21,165 chest X-ray images	Normal, COVID-19, Pneumonia Lung Opacity	Deep/Transfer learning models	Decent ternary classification accuracy Validation: 95.63%	Ternary classification, imbalanced data
[34]	ChestX-ray8 dataset with 1125 X-ray images	Binary (COVID-19/Healthy) Ternary (COVID-19, Healthy/Pneumonia)	Deep learning models DenseNet169 MobileNet	Best binary classification accuracy 98.54% for binary and 91.11% for ternary	Only binary and ternary classification (not four classes)
[35]	Public dataset with chest X-ray images	Binary (no finding/pneumonia) Multivariate (COVID-19/No findings/Pneumonia)	Deep Learning, Transfer Learning models	Comprehensive study with several experiments and decent accuracy Binary (91.5%) Multivariate (91.11%)	Only binary and ternary classification (not four classes), little difference in accuracy metric

**Table 2:** Number of images in each class.

Class	Number of Images
COVID-19	3616
No-Findings (Normal)	10,192
Pneumonia	4273
Tuberculosis	3500

**Table 3:** Parameters used in this study.

Layer (Type)	Output Shape	Parameters
Rescaling	(None, 300, 300, 3)	0
Conv2d	(None, 300, 300, 16)	448
Max_pooling2d	(None, 150, 150, 16)	0
Conv2d	(None, 150, 150, 32)	4640
Max_pooling2d	(None, 75, 75, 32)	0
Conv2d	(None, 75, 75, 64)	18,496
Max_pooling2d	(None, 37, 37, 64)	0
Conv2d	(None, 37, 37, 128)	73,856
Max_pooling2d	(None, 18, 18, 128)	0
Conv2d	(None, 18, 18, 256)	295,168
Max_pooling2d	(None, 9, 9, 256)	0
Dropout	(None, 9, 9, 256)	0
Flatten	(None, 20736)	0
Dense	(None, 128)	2,654,336
Dense	(None, 4)	516

**Table 4:** Classification report for the proposed model.

Class	Precision	Recall	F1-Score
COVID-19	97.00%	96.27%	96.63%
NO-FINDINGS	98.72%	99.35%	99.04%
PNEUMONIA	99.89%	99.66%	99.77%
TUBERCULOSIS	98.90%	98.10%	98.50%

**Table 5:** Inference results.

	Predicted				
Actual	COVID-19	No-Findings	Pneumonia	Tuberculosis	Correctly Classified
COVID-19	22	3	0	0	22/25
NO-FINDINGS	0	25	0	0	25/25
PNEUMONIA	0	0	25	0	25/25
TUBERCULOSIS	0	0	0	25	25/25

**Table 6:** Comparison with state-of-the-art.

Study	Dataset Size	Diseases/ Classes	Techniques Used	Average Accuracy
Bhandari et al. [28]	Public dataset with 7132 chest X-ray images	COVID-19 Pneumonia Tuberculosis No-Finding	Deep Learning and XAI	Testing: 94.31 ± 1.01% Validation: 94.54 ± 1.33%
Venkataramana et al. [29]	Public dataset with 14,693 chest X-ray images	COVID-19 Pneumonia Tuberculosis No-Finding	SMOTE and Deep Learning	95.7% without Balancing 96.6% with Balancing
Bashar et al. [32]	Public dataset with 21,165 chest X-ray images	Normal, COVID-19, Pneumonia Lung Opacity	Deep learning models	Validation: 95.63%
Nasiri et al. [34]	ChestX-ray dataset with 1125 X-ray images	Binary (COVID-19/Healthy) Ternary (COVID-19, Healthy/Pneumonia)	Deep learning models DenseNet169 MobileNet	98.54% for binary and 91.11% for ternary
Liu et al. [35]	Public dataset with chest X-ray images	Binary (no finding/pneumonia) Multivariate (COVID-19/No findings/Pneumonia)	Deep Learning, Transfer Learning models	Binary (91.5%) Multivariate (91.11%)
Proposed Technique	Public dataset with 21,581 chest X-ray images	COVID-19 Pneumonia Tuberculosis No-Finding	Deep learning models (CNN)	Validation: 98.72%

## Data Availability

The dataset is available with the corresponding author and can be provided on a reasonable request.
